# Entrepreneurship in Latin America from a sociological perspective: a systematic literature review (2020–2025)

**DOI:** 10.3389/fsoc.2026.1806935

**Published:** 2026-06-04

**Authors:** Carlos José Monroy-Barrero

**Affiliations:** 1 Facultad de Ciencias del Comportamiento, Universidad de La Sabana, Chía, Colombia; 2 School of Human Sciences, Universidad del Rosario, Bogotá, Colombia

**Keywords:** entrepreneurship, individuation process, Latin America, mixed methods, social ordeal

## Abstract

**Introduction:**

In Latin America, entrepreneurship has traditionally been analyzed through administrative and economic lenses, often overlooking its sociological implications. This study re-conceptualizes entrepreneurship as a social ordeal analyzing how individuals navigate structural constraints, labor informality, and neoliberal mandates within the framework of the sociology of individuation.

**Methods:**

Following PRISMA 2020 guidelines, a Mixed Methods Systematic Review (MMSR) with a convergent design was conducted on a corpus of 74 articles (2020–2025). Quantitative descriptive analysis (SPSS) and qualitative interpretative analysis (MAXQDA) were integrated to triangulate thematic trends, geographical distributions, and discursive patterns.

**Results:**

The findings reveal an epistemological diversity in regional production: Brazilian literature critiques the “uberization” of work, while Mexican research emphasizes resilience and adaptation. Colombia recognizes entrepreneurship as subsistence perspective. A predominant trend toward group-level analysis identifies entrepreneurship as a collective survival strategy rather than individual innovation. Furthermore, entrepreneurship acts as a social escape valve of the workforce in informality, where autonomy is a forced management of precariousness.

**Discussion:**

The study proposes understanding entrepreneurship as a social ordeal, generating a model to increase the comprehension of the phenomenon in Latin America, where individual agency exists in permanent tension with structural inequalities. The model illustrates that this ordeal is unequally distributed by non-elective attributes (class, gender, and geography) and is socially produced through varied regional lenses, from functionalist resilience to critical perspectives. Under neoliberal rationality, the “entrepreneurial spirit” serves as a culturally represented discourse that transfers systemic risk to the subject, legitimizing self-exploitation as a form of “freedom.” Ultimately, the ecosystem forces complex individuation processes, where the subject must tactically rely on informal support to negotiate their existence against a precarious reality.

**Conclusion:**

Entrepreneurship in Latin America is a manifestation of mandatory individuation. To mitigate the solitude of this structural social ordeal, public policies must transition from promoting individual competition to strengthening collective support networks and social guarantees that spread the risks linked to motivations originating in need and entrepreneurial ecosystems in development.

## Introduction

In Latin America, a complex interplay of social, political, and cultural dynamics is inextricably linked to structural inequality. This situation stems from labor informality, lack of access to essential services, violence, low competitiveness, and palliative state responses. Between 2020 and 2025, these dynamics were significantly exacerbated by the effects of COVID-19 pandemic. Within this framework, entrepreneurship emerged as one of the primary subsistence strategies for individuals. During 2021 and 2022, countries such as Ecuador, Chile, and Colombia reported that between 25 and 30% of their adult population was starting a business; in 50% of these cases, the primary driver was the lack of employment resulting from the pandemic’s impact (Global Entrepreneurship Monitor, 2025).

The impact of COVID-19 on Latin American entrepreneurial ventures triggered a structural crisis, causing 53% of them to cease commercial operations, with a more severe effect on younger enterprises. This paralysis weakened the organizational climate of 49% of workforces and exposed a critical digital divide, as only 37% successfully implemented remote work. Furthermore, the ecosystem faced institutional vulnerability, with 68% lacking adequate emergency support services and 64% experiencing a critical contraction in funding sources, thereby severely compromising their long-term viability (Santamaria Velasco et al., 2021). According to the ILO (ILO, 2024), 51% of Latin American workers operate under conditions of informality. Furthermore, over 70% of the region’s productive units are micro-enterprises (1 to 10 employees) or self-employed individuals, many of whom lack social protection or access to formal financing. ECLAC (ECLAC, 2024) warns that these dynamics are heightened in contexts of extreme inequality: on average, the wealthiest 10% of the Latin American population concentrates 55% of the total income. In this regard, the (OCDE, 2023) argues that regional nations must transform their productive structures to become more sustainable, inclusive, and resilient. This transformation requires fostering the development of startups, improving coordination across government levels, and closing gaps in infrastructure and financing.

Against this backdrop, the present research conducts a systematic literature review to address the following questions: What are the main challenges facing entrepreneurship in Latin America? And how have these challenges been academically addressed through a sociological lens between 2020 and 2025? Analytically, this proposal is grounded in understanding the dynamics and tensions between the individual, their capacity for agency, and the social structure in which they are embedded.

The selection of Latin America as the unit of analysis responds to the need to address a phenomenon that, while global, is profoundly mediated by contextual conditions. As argued by Cervelló-Royo et al. (2025), focusing on this region allows for an understanding of the heterogeneity of cultural and historical contexts by analyzing how specific variables impact the entrepreneurial ecosystem. Consequently, the region presents a unique configuration where institutional fragility and structural informality dictate entrepreneurial trajectories that differ substantially from the logic observed in developed economies.

## Theoretical framework

### Sociology of individuation

Sociology is the scientific discipline that studies social relations, structures, practices, and institutions that shape life in society (Giddens, 2009). In this sense, it allows us to understand how individual actions are conditioned by broader social contexts, such as cultural norms, social classes, gender, power, or political and economic institutions. Consequently, the sociology of entrepreneurship does not focus solely on the economic act of “starting a business”; rather, it explores how entrepreneurial subjects are symbolically constructed, negotiate identities, exercise agency, and confront social challenges within specific contexts. The discipline strives to develop stable models that explain social reality, shaping its challenges to intervene in them effectively.

The sociology of modernity combines the ability to construct global representations of studied phenomena ideal types that establish the distance between the awareness of what must change and the asymmetry that exists with reality. According to Martuccelli (2013a,b), in any analyzed phenomenon, it is essential to maintain the link between the individual and the structural dimensions of society. Likewise, the study of singularity must highlight its specific socio-historical localization; a certain stability in the relationship between the individual and the structure must be recognized. The process must allow for a renewed idea of agency that accounts for the “work on the self” carried out by everyone.

In this vein, a phenomenon such as the entrepreneurial process can be framed as a social ordeal to which individuals are subjected. “Ordeals are historical challenges, socially produced, culturally represented, and unevenly distributed, which individuals are compelled to face within a structural process of individuation” (Martuccelli (2013a,b), p. 215). This concept will guide the main results and discussion of this article.

### Toward a sociology of entrepreneurship

Sociological explanations related to entrepreneurship express that human behavior is complex and unpredictable. It cannot be explained by a model that tends to simplify or omit behavioral aspects such as emotions, cultural beliefs, or cognitive factors like the need for achievement elements that, in short, may not fit well within an economic model. In the authors’ words: “studies examining non-economic factors have highlighted the fact that the mere provision of economic inputs may not guarantee success in entrepreneurial ventures” (Terán-Yépez and Guerrero-Mora, 2019, p. 11). Therefore, reading that delves into other areas surrounding the activity of undertaking must be promoted, for instance, the motivations for doing so, or for continuing to try even after failure.

### Entrepreneurial ecosystem

The definition of the concept of entrepreneurship is not unique, nor does it belong to a particular academic field. According to Cuellar Chavez (2025), in the 21st century, one of the challenges lies in characterizing the entrepreneur beyond the traditional traits that describe a business. These traits generally limit the entrepreneur to a capitalist interest, when entrepreneurs are people seeking more than economic profit.

Along these lines, entrepreneurship must be analyzed through ecosystems understood as the “interaction of political, social, economic, cultural, and environmental aspects that allow for the development of entrepreneurial activity in a given region” (Marín-Cardona and Cuartas-Torres, 2022, p. 1). In Latin America, this interaction does not occur harmoniously; on the contrary, deep-rooted processes such as corruption, internal violence, illegal economies, forced displacement, and the region’s lack of infrastructure, among others, force the ecosystem to operate under a logic of subsistence. Thus, the pillars of the ecosystem do not only support business creation but also shape a forced individuation, where the subject must compensate for the deficiencies of a political and economic environment that offers neither clear nor stable guarantees through informal networks and personal support.

### Opportunity vs. necessity-based entrepreneurship

The distinction between opportunity based and necessity-based entrepreneurship constitutes a fundamental analytical axis for understanding the relationship between social context and the motivation to start a business. As noted by Terán-Yépez and Guerrero-Mora (2019), while “pull” factors associated with proactive agency, the search for autonomy, and innovation predominate in advanced economies, “push” factors prevail in Latin America. In the region, entrepreneurship emerges primarily as a survival response to the precariousness of the labor market. This landscape places Latin America, Africa, and Southeast Asia within an ecosystem of high entrepreneurial activity but low innovative intensity and low business sustainability. In these contexts, the creation of productive units acts more as a subsistence mechanism than as a driver of economic development or quality employment generation.

Under this logic, necessity-based entrepreneurship should not be interpreted solely as an economic metric, but as a manifestation of the process of individuation and the transfer of risk toward the individual. The predominance of necessity-based motivation in the region reveals how institutional deficiencies and macroeconomic instability force the individual to assume total responsibility for their livelihood, transforming structural vulnerability into a demand for personal management. Nevertheless, it is imperative to recognize that this boundary is porous; an entrepreneur’s agency can allow for a transition where an initiative born of urgency is transformed, through the identification of viable alternatives, into an opportunity-based project. Integrating this perspective allows for a deeper understanding of how an unequal economic structure conditions the motivations to undertake.

## Methodology

This article conducts a systematic literature review following the PRISMA 2020 (Preferred Reporting Items for Systematic Reviews and Meta-Analyses) guidelines. The objective is to identify, classify, and analyze the academic production about entrepreneurship in Latin America from a sociological perspective. The choice of the PRISMA framework stems from its capacity to promote transparency and comprehensiveness throughout all stages of the review process from the formulation of the search strategy to the selection, evaluation, and synthesis of the included studies. This methodological rigor is particularly relevant in the social sciences, where the conceptual and methodological heterogeneity of entrepreneurship studies requires clear and consistent criteria for pertinent analysis. In this regard, the use of PRISMA enabled the construction of a robust review based on replicable procedures and explicit inclusion and exclusion criteria (Page, 2021).

### Protocol and registration

The protocol was established prior to the commencement of the search process and was documented in a file that included: a clear definition of the research question; criteria for the inclusion and exclusion of studies; selected databases; the search strategy (including key terms and Boolean operators); screening and filtering procedures; the design of the data extraction matrix; and the analysis plan, including the assessment of risk of bias in the articles. The advanced process is described below.

The search strategy was implemented between April and August 2025, covering five academic sources: Web of Science, SciELO, Scopus, Dialnet, and Google Scholar. Tailored search strings were applied to each database, utilizing a combination of Boolean operators and key terms such as: *(entrepreneurship OR entrepreneur OR entrepreneurism) AND [Latin America OR (country names)] AND (sociology OR subjectivity OR self-employment OR gender OR development OR social OR capitalism)*.

#### Inclusion criteria

- *Period*: 2020–2025.
- *Languages*: Spanish, Portuguese, and English.
- *Document type*: Published scientific articles.
- *Access*: Open-access full texts.
- *Disciplines*: Sociology or related fields within social studies.

**Figure 1:**
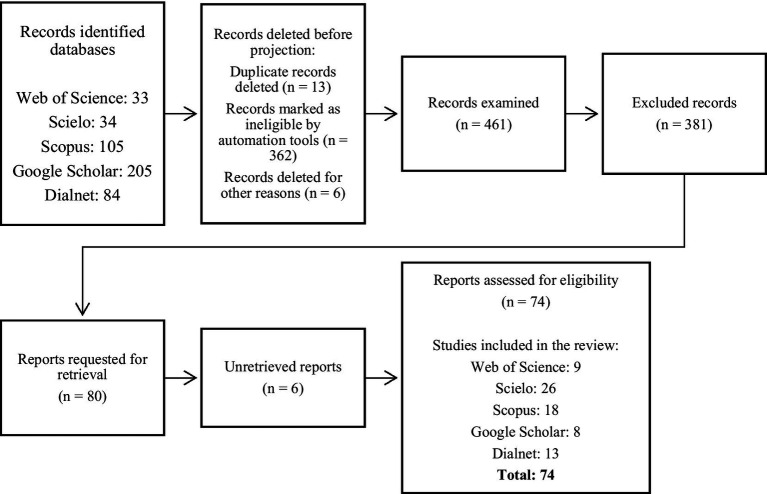
PRISMA 2020 flow diagram for the systematic review. Source: own elaboration based on Page (2021).

The search equation was not designed to establish a synonymous relationship between descriptors but rather as a multidimensional capture strategy aimed at ensuring the sensitivity of the retrieval process. Under this logic, the OR Boolean operator was employed to group terms that, in sociological literature, represent concurrent dimensions of the phenomenon. While the term self-employment allowed for the capture of research focused on labor market transformations and informality, the descriptor gender ensured the inclusion of studies analyzing entrepreneurship as a practice shaped by structures of power and inequality. Finally, the term sociology functioned as a disciplinary affiliation filter. This combination allowed the study to transcend purely administrative visions of entrepreneurship, consolidating a documentary corpus that recognizes the object of study as a situated and structurally conditioned social practice Table 1, Figure 1.

**Table 1:** Inclusion and exclusion criteria.

Inclusion criteria	Exclusion criteria
Articles about Latin American countries	Articles about other geographic regions
Main approach from a sociological perspective	Articles with a focus only on technical, economic or business development
Published between 2020 and 2025	Production prior to 2020
Articles with full access to the text.	Articles without the possibility of accessing the full text.

Source: own elaboration.

### Construction of the analysis matrix

To organize and systematize the information extracted from the selected articles, an analysis matrix was constructed, structured into four thematic blocks. The first block, Bibliometric Data, included information such as authors, year, journal country, database, and keywords. The second block, Case Study Data, identified the country analyzed, economic sector, territorial scope (local, regional, national, or transnational), and target population (men, women, or both). The third block, Methodological Dimensions, collected the type of study (theoretical or empirical), methodological approach (qualitative, quantitative, or mixed), sociological level of analysis (individual, group, or structural), the analyzed economic sector, and the data sources used. Finally, the fourth block addressed Sociological and Discursive Dimensions, coding definitions of entrepreneurship, ideological positions, main findings, declared objectives, and primary results. This matrix enabled the integration and triangulation of the study’s quantitative and qualitative approaches, facilitating a relational and critical understanding of the entrepreneurship phenomenon.

It should be noted that a glossary of terms was developed to code the articles under uniform parameters regarding both theoretical and methodological definitions. Likewise, a quality assessment of the reviewed articles was conducted through academic peer review.

The database was manually created from the 74 articles selected as the final result of the search and filtering process according to PRISMA criteria. For each article, the full text was individually downloaded in PDF format, ensuring complete access to the content. Subsequently, a thorough reading of each article was performed, combined with the assisted use of Artificial Intelligence (AI) tools, specifically Scholar GPT, which facilitated the preliminary identification of objectives, findings, key categories, and relevant citations. This work was carried out by the researcher, ensuring that decisions regarding coding, interpretation, and classification of information were made through human analytical judgment and were not exclusively delegated to AI.

### CASP analysis

As a complementary process with the information consolidated in the analytical matrix, the research evaluated the quality of the articles. According to the Critical Appraisal Skills Program (Critical Appraisal Skills Programme, 2018), the analysis of articles selected for a systematic review must include a quality assessment to establish their validity, rigor, and utility based on 10 specific criteria. The results are presented below.

The application of the CASP protocol allows for the conclusion that the sample of 74 articles selected for this systematic review possesses a high level of rigor and scientific relevance, providing validity to the analyses presented Table 2.

**Table 2:** CASP analysis.

Axle	Criteria	Quality evaluation of articles
Validity	Clear objectives	The 74 articles included, that is, 100%, clearly declare their research commitments. In particular, this study analyzes in its entirety the proposed research objectives, as well as the methodology.
	Appropriate methodology	
	Appropriate methodological design	
Rigor	Participant sampling/selection	Of the 74 articles, 51 are empirical, that is, 69% of these declare clear and rigorous procedures of fieldwork.
	Data collection	
	Reflexivity	This criterion is especially developed in the theoretical studies of 23 of the studies, 31% that deepens the phenomenon of study from a critical perspective.
	Ethics	It is a component that should be strengthened, especially in 69% of the articles, since it is usually presented in a technical way (informed consent) but does not address in depth how the relationship with participants develops during the research process, as well as processes of return of results, among others related to the ethical relationship with people.
	Rigorous data analysis	The 74 articles present a coherent data analysis in relation to the objectives set, as well as from the data collection techniques, the volume of information collected and the different types of analysis according to the method implemented.
Usefulness	Clear findings	They are presented in all 74 articles and are analyzed in this study as the central contributions they provide to the understanding of the phenomenon studied.
	Value of research	100% of the studies present relevant contributions to the study phenomenon that are deepened in the results section.

Source: own elaboration.

### Data processing

This research was grounded in a Mixed Methods Systematic Review (MMSR) design with a convergent approach. Following Ghiara (2020) and Woolley (2008), this design enables the systematic integration of quantitative and qualitative data within a single synthesis phase.

First, a quantitative descriptive analysis was developed using SPSS software, coding variables such as publication year, case study country, database, methodological approach, and sociological level of analysis. This data allowed for the identification of thematic trends and patterns. Second, an interpretative qualitative analysis was applied using MAXQDA software, which enabled the coding of articles based on interpretative analytical categories. Open and axial coding techniques were employed, alongside word cloud analysis, semantic associations, and co-occurrence maps, facilitating the identification of discursive patterns, ideological positions, and meanings attributed to entrepreneurship. This methodological combination allowed for the triangulation of results, enriching the understanding of the phenomenon and strengthening the analytical validity of the findings.

### Risk of bias

In accordance with the PRISMA 2020 guidelines, any systematic review must include a rigorous assessment of the risk of bias in the included primary studies to evaluate the internal validity of the evidence and enhance transparency in the interpretation of results. “The risk of bias in the results of each included study should be assessed using appropriate criteria; these criteria will vary according to the study design” (Page, 2021, p. 4). Consistent with this requirement, three axes are analyzed: the origin of the analyzed studies; the type of study and its methodological approach; and the analytical proposal from a disciplinary perspective by study type.

### Database selection

The selection of databases was carried out with the aim of ensuring a broad, rigorous, and representative coverage of academic production on entrepreneurship in Latin America from a sociological perspective. Databases with high levels of international indexing, such as Web of Science (9 articles) and Scopus (18 articles), were included to ensure the visibility and impact of the publications, as well as their scientific relevance. On the other hand, platforms such as SciELO (26 articles) and Dialnet (13 articles) were incorporated to provide access to studies published in high-quality regional journals, which are particularly relevant within the field of Latin American social sciences. Finally, Google Scholar (8 articles) was utilized as a complementary resource to identify potentially relevant studies not indexed in other platforms. This strategic combination of databases allowed for a balance between academic rigor and epistemological and territorial diversity, which was fundamental for organizing the final sample of articles.

### Methodology of articles

**Figure 2:**
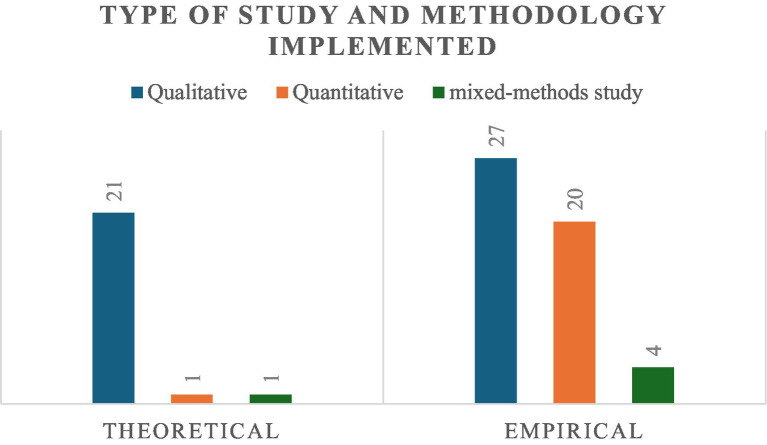
Total articles included by type of study and methodology implemented. Source: own elaboration.

Thus, the 74 included articles were analyzed based on two primary criteria: the type of study (theoretical or empirical) and the methodology employed (qualitative, quantitative, or mixed). This dual-axis approach allowed for the identification of common research configurations which, beyond their technical quality, present certain analytical limitations or interpretative biases. For instance, although 51 of the studies were empirical, the majority focused on qualitative approaches (48) aimed at describing situated experiences. In contrast, quantitative studies (21) tended to adopt functionalist or variable-centered frameworks without sufficiently incorporating structural or relational dimensions. Finally, only 5 studies applied mixed methodologies, revealing a narrow margin for integrating the contextual depth of qualitative research with the generalizability of quantitative data Figure 2.

### Ethical considerations

This study approached ethical challenges that must be highlighted: algorithmic bias, selection bias, and language restrictions. The use of AI tools was mediated by constant epistemological vigilance to avoid the reproduction of classification biases regarding the definition of entrepreneurship, particularly from purely economic perspectives. Likewise, it is acknowledged that reliance on indexed databases and the preeminence of English in global academia may lead to the omission of voices from the periphery or other non-academic forms of knowledge. Future research should integrate gray literature and local knowledge networks to capture the full plurality of the entrepreneurial ecosystem in the Global South.

In the same way, Artificial Intelligence specifically Scholar GPT was used exclusively as a technical support tool during various phases of the systematic review process. Its use was limited to optimizing search strings for academic databases, performing preliminary searches, validating inclusion criteria, assisting in thematic classification, and reviewing abstracts. Furthermore, it served as an assistant in finalizing the analysis matrix without intervening in its design, variable selection, or the final coding of analysis categories, which were subsequently processed in SPSS and MAXQDA. AI was also utilized to suggest improvements in academic writing and to ensure compliance with the methodological standards established by PRISMA 2020. All methodological, theoretical, analytical, and interpretative decisions were made autonomously by the researcher, ensuring that critical judgment, academic reflection, and human control governed every stage of the study. Additionally, principles of ethical citation, academic integrity, and source traceability were respected in accordance with current APA standards.

## Results

As a general context for this section, an analysis of the keywords declared in the 74 articles included in this review is presented. Through an analysis using MAXQDA software, it allows us to establish the most recurrent categories in the studies. As expected, the term “entrepreneurship” occupies a central position, yet it is accompanied by other notions such as “social,” “community,” “informal,” and “resistance.” This suggests a sustained interest in understanding the phenomenon beyond traditional business approaches, recognizing its inscription in contexts of inequality, exclusion, and innovation.

Similarly, the prominent presence of terms such as “neoliberalism,” “education,” “gender,” “development,” “public policy,” and “capital” provides a comprehension about the diversity of theoretical perspectives and analytical lenses that shape this field of study. This combination of categories reveals a latent tension between visions that conceive entrepreneurship as a mechanism for individual innovation and empowerment, and those that interpret it as an adaptive response to structural conditions of exclusion. In this sense, the key words not only synthesize the dominant vocabulary in recent academic production but also highlights the disputes over the meanings of entrepreneurship: as public policy, as a situated social practice, and as a contested category between the economic, political, and cultural spheres.

To further delve into the findings, the results section is divided into three parts: First, The countries’ academic production. Second, sociological perspective analysis. Third, an understanding of the challenges facing entrepreneurship in the region.

### The countries’ academic production

The evolution of publications on entrepreneurship framed or produced were increasing through the time frame analyzed: 2020 (11 articles) 2021 (14 articles) 2022 (12 articles) 2023 (18 articles) 2024 (17 articles) and 2025 (2 articles). A sustained growth in academic interest in the subject is observed, with a notable peak in 2023 and 2024. In this line it is important to note that the production is higher in the follow countries: Mexico (16 articles), Brazil (14 articles), Ecuador (14 articles), and Colombia (10 articles).

First topic to present is about the key conceptual contributions from these regional blocks that show different approaches to the understanding of this phenomenon. In Mexico, there is a strong emphasis on analyzing the challenges facing the consolidation of entrepreneurial activity: “The lack of personal resources, the need to balance studies and employment, and initial fears are the primary causes of project abandonment, even within high-quality incubators” (Barroso Tanoira et al., 2020, p. 16).

This may be linked to the social and economic consequences of the COVID-19 pandemic, which amplified discussions regarding subsistence strategies, labor informality, and the need for economic reconstruction in contexts of high inequality. As described in some of the studies, particularly in Mexico: “Female entrepreneurs redefined their businesses during the pandemic, incorporating digital strategies and community support networks” (Hernández and Flores, 2022, p. 8).

Beyond the COVID-19 milestone, the analyzed academic production generally suggests a disciplinary opening toward critical, intersectional, and territorial approaches to entrepreneurship, responding to the complexity of contemporary challenges faced by Latin American countries. For example, Cornejo Pérez (2020) explores how, in neoliberal contexts, hip-hop youth find a symbolic and social outlet within the cultural market through cultural entrepreneurship. In the same vein, Nogueira (2023) argues that entrepreneurship acts as a neoliberal governance device that legitimizes social atomization and competitiveness as a social norm.

In the case of Ecuador, for instance, Chusin Ayala (2024) highlights the importance of access to financing systems: “Accessibility to microcredit is the most influential factor, followed by social cohesion, growth, and survival. Microcredit is key to community resilience” (Chusin Ayala, 2024, p. 17). In the articles from Brazil, stances are framed that critically question the ideological construction of the entrepreneur as a neoliberal and urban way of life (Amorim, 2021).

Finally, in Colombia, we can highlight the study by (Pacheco-Fuentes et al., 2023) which analyzes the tension between individual interest and social contribution is further evidenced in empirical studies within the region, such as the research conducted on Small and Medium-sized Enterprises (SMEs) in Barranquilla, Colombia. This study reveals that, in the wake of the economic crisis and job losses triggered by the COVID-19 pandemic, the entrepreneurial landscape is dominated by a “business entrepreneur” profile primarily focused on achieving personal benefits. Despite the systemic call for these organizations to act as drivers of social and economic development, there is a notable absence of practices associated with social entrepreneurship or collective value creation. This finding suggests that in contexts of high structural uncertainty, the subject’s agency is funneled toward individual survival and the management of personal vulnerability, leaving the ambition of social transformation as an unfulfilled expectation within the neoliberal ecosystem.

**Figure 3:**
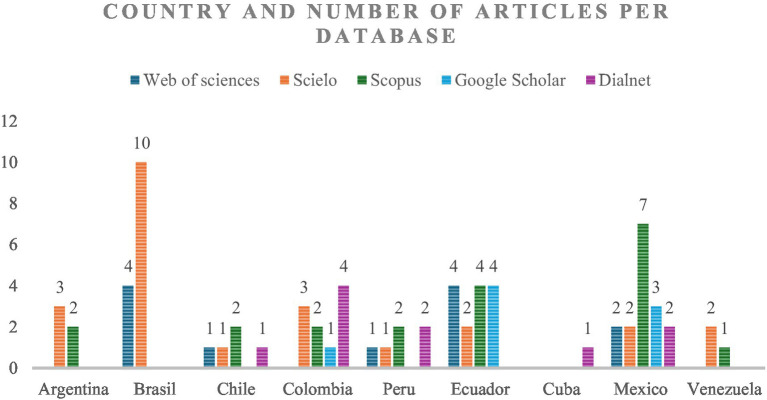
Country and number of articles per database. Source: own elaboration.

A second tendence is linked with the analysis of the origin of the studies by case-study country and database (Figure 3) to know about the Scope and impact on the academic debate on production, this reveals a significant inequality in the visibility and dissemination of academic knowledge regarding entrepreneurship in Latin America.

Brazil and Mexico stand out as the countries with the highest number of identified articles, maintaining a solid presence in both regional databases (such as SciELO and Dialnet) and high-impact international ones (Web of Science and Scopus). This diversity suggests a consolidated academic infrastructure and greater integration into global editorial networks.

In contrast, countries such as Ecuador, Colombia, and Argentina show a visible production, but it is primarily indexed in regional databases, which may limit its impact in other regions. Furthermore, Chile, Peru, Cuba, and Venezuela exhibit reduced representation, concentrated in open-access databases like Google Scholar, highlighting limitations in terms of resources, editorial policies, and scientific indexing. These gaps in the distribution of studies not only reflect asymmetries in academic production but also hinder the possibility of including diverse voices and territorial experiences in the construction of sociological knowledge about entrepreneurship in the region.

**Figure 4:**
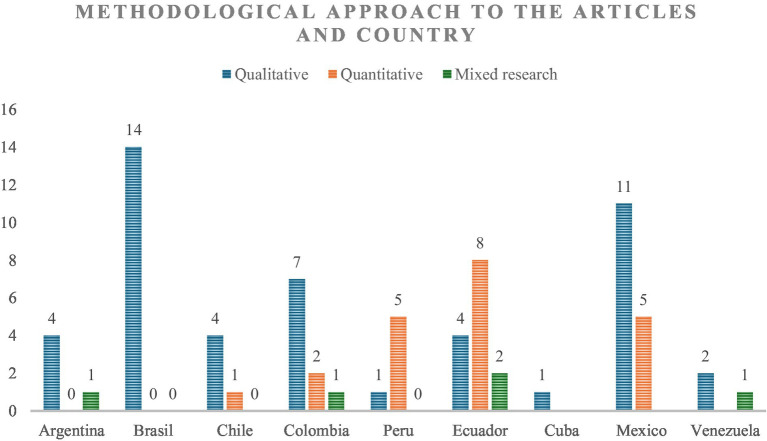
Methodological approach to the articles and the country. Source: own elaboration.

As third topic, it is important to highlight that a qualitative approach predominates in the region. Notably, countries such as Brazil stand out, as the totality of identified studies from this context employ this methodology Figure 4.

Among the reasons for this trend, we can highlight that most studies seek to explore entrepreneurs’ narratives based on their own lived experiences. Furthermore, there are often studies of specific populations or concrete locations that allow for a situated approach to the phenomenon. For example, a case study from Chile (Krell Rivera, 2020) highlights that the creative appropriation of the “development with identity” discourse allows indigenous leaders to generate their own regulations for tourism and the protection of their territory.

In all qualitative studies, data collection techniques associated with this perspective are implemented. Interviews in their various modalities (focus groups, semi-structured, in-depth, and biographical) are a recurrent primary source, often complemented by participant observation and documentary analysis. The latter includes a review of academic literature, reports, articles, institutional documents, and public policies.

Surveys constitute another fundamental data source, applied to diverse groups such as university students, micro-entrepreneurs, customers, and producers, and frequently validated through rigorous statistical analysis. Such is the case of a study developed in Mexico based on a survey of 1,060 micro-entrepreneurs, which concludes that: “Gender moderates the effect of schooling, the manufacturing sector, and the motivation to pursue a career, as well as the willingness to change activities; micro-enterprises led by women show a lower probability of continuity when operating in manufacturing” (Ortiz-Rodríguez, 2020, p. 28). Similarly, most quantitative studies utilize large-scale secondary data, such as those from the Global Entrepreneurship Monitor (GEM) or official statistical sources from each country.

The reviewed studies prioritize a deep understanding of trajectories, discourses, and forms of resistance, utilizing methodologies that allow for the exploration of the meanings that actors attribute to their practices. In turn, this trend reveals common findings, primarily resulting from interviews: “The lack of personal resources, the need to balance studies and employment, and initial fears are the primary causes of project abandonment, even within high-quality incubators” (Barroso Tanoira, 2020, p. 16).

The methodological analysis gives a clear gap identification, the importance to delimitate research mixed approach to strength the understanding of entrepreneurship and these dynamics. This is fundamental considering that entrepreneurship is in the intersection of structure and agency, and this invites them to approach the quantitative to get the structural processes and the qualitative to get the individual processes.

### Sociological analysis

The 74 articles included in the review, 32 focuses on the group level, 29 on the structural level, and 13 on the individual level. The marked preference for the group-level approach indicates a tendency to understand entrepreneurship as a collective experience, sustained by networks, bonds of reciprocity, or community dynamics, especially in scenarios characterized by exclusion and precariousness Table 3.

**Table 3:** Sociological levels of analysis in the reviewed studies.

Level of analysis	Definition	Number of articles
Individual	Focused on the agency of the individual entrepreneur: their motivations, aspirations, decisions, and trajectories. This level analyzes how individuals interpret their reality and act within their contexts, highlighting their reflexive capacity.	13 Empirical
Group	Focused on social relations, support networks, community dynamics, and collective action. It explores how entrepreneurship is shaped through interaction with other actors, such as families, associations, or collectives.	3 Theoretical/29 Empirical
Structure	Addresses macro conditions that affect or facilitate entrepreneurship: public policies, institutions, inequalities of class, gender, and ethnicity, and the economic system. It allows for an understanding of the social framework that structures entrepreneurial practices.	20 Theoretical/9 Empirical

Source: own elaboration.

Theoretical studies predominantly focus on the structural level, highlighting a gap in the theorization of the phenomenon based on empirical evidence. Conversely, empirical research shows that, while there is an abundance of studies on entrepreneurial practices and experiences, few successfully link these realities to the broader social structures that shape them.

In summary, this systematic review reveals that, although there is a growing production of empirical studies on entrepreneurship in Latin America, many remain anchored in individual or group levels of analysis. They fail to achieve profound articulation with the social structures that configure historical inequalities. This misalignment does not imply a hierarchy among methodological approaches; rather, it represents a limitation in explanatory capacity when the experience of entrepreneurs is dissociated from the structural frameworks that either enable or restrict it.

### Territorial focus and level of analysis

**Figure 5:**
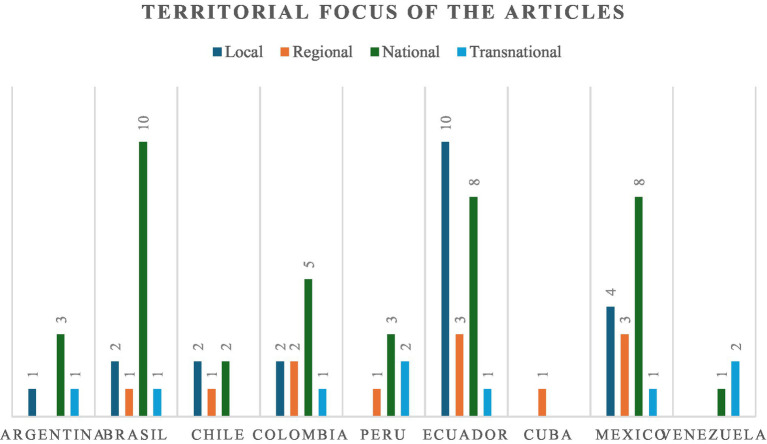
Territorial approach of the articles. Source: own elaboration.

In sociology, analysis across different levels or systems allows for an understanding of how social dynamics are articulated within various geographical and institutional settings. According to Glick Schiller (2016), the concepts of the local, national, and transnational should be understood not as fixed hierarchical levels, but as interconnected spaces where practices, identities, and power structures are configured. Thus, a multiscale perspective makes it possible to observe how local conditions for entrepreneurship are influenced by national policies or global flows of capital, technology, and ideology Figure 5.

The analysis of the reviewed studies reveals the existence of multiple levels of analysis surrounding the phenomenon of entrepreneurship in Latin America, reflecting various scales of social interaction. At the local level, entrepreneurship manifests in situated experiences within specific communities, neighborhoods, or territories, where practices linked to informal economies, family networks, or identity processes prevail. Examples such as rural cooperatives or neighborhood markets demonstrate how these ventures contribute to income generation and community support, even in contexts of high precariousness, as illustrated by Martínez (2020) when describing denim workshops as forms of family entrepreneurship under structurally limited conditions.

At the regional scale, some studies address dynamics that span provinces or departments, highlighting public policies for territorial development, local productive clusters, and urban–rural disparities for instance, emphasizes how female leadership in indigenous communities has sustained economic processes and reinforced social cohesion.

On the national plane, the analysis focuses on the role of the State through regulatory frameworks, development policies, or institutional discourses on entrepreneurship. This includes specific laws for MSMEs (Micro, Small, and Medium Enterprises), differentiated tax systems, or innovation incentive programs, as noted by Mellado Ibarra and Sánchez Tovar (2023), who establishes that the Mexican entrepreneurial ecosystem exhibits strengths in infrastructure and human capital but weaknesses in financing and technological innovation.

Finally, the transnational level allows for a more structural reading of the phenomenon, considering the relationships between entrepreneurship, migration, international cooperation, and processes of economic and cultural globalization. In this sense, authors such as Presta (2022) conceptualize entrepreneurship as a governance device inherent to contemporary neoliberalism, which redefines the management of labor and poverty through logics of individual self-management. Despite this diversity of approaches, a predominant trend toward regional and national studies is observed, while local and transnational perspectives remain scarce. This theoretical and methodological omission limits the understanding of entrepreneurship as a multiscale and situated practice.

Therefore, it is essential to incorporate perspectives that articulate the local experiences of entrepreneurs with transnational flows of capital, knowledge, and technologies to enrich the sociological field of entrepreneurship in Latin America.

### Challenges of entrepreneurship in the region

What are the research objectives proposed in the analyzed studies? This question is fundamental to understanding how the challenges and hurdles of entrepreneurship in Latin America are framed from an academic perspective. When analyzing the semantic association network built from the objectives of the 74 included studies, a clear intention to analyze and understand the entrepreneurial phenomenon in relation to its social, political, and economic context is observed. Far from a merely technical or business-oriented vision, these studies tend to approach entrepreneurship as a situated social practice, influenced by power structures, individual trajectories, and relational dynamics.

**Figure 6:**
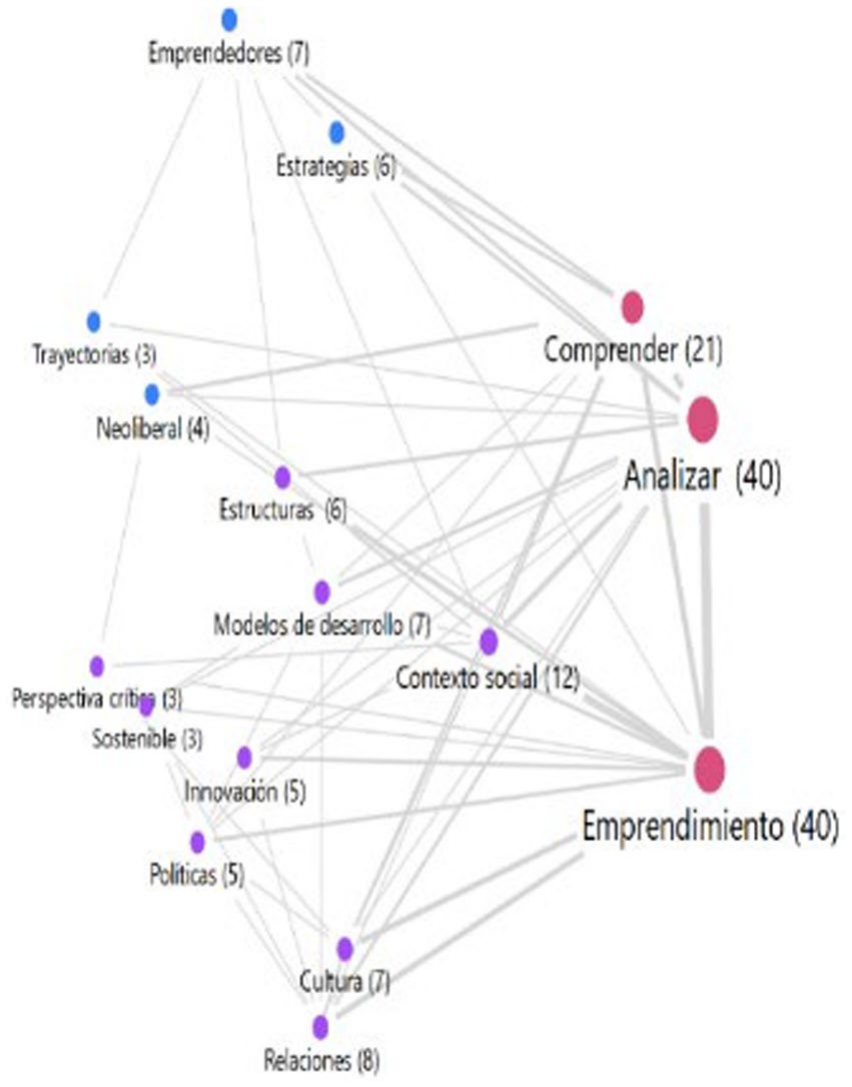
The semantic association of general objectives exposed in the articles analyzed. Source: own elaboration.

The emphasis on notions such as development models, relationships, structures, social context, and critical perspective reflects a common concern with problematizing the structural and institutional conditions that limit or condition entrepreneurial action. Thus, the challenges of entrepreneurship in the region are not reduced to mere management or financing barriers; instead, they are framed within broader processes of inequality, informality, and tensions with neoliberal logic. This necessitates more comprehensive, reflexive, and contextualized approaches Figure 6.

The semantic association network demonstrates that the most frequent and central words in the research objectives are “Analyze,” “Entrepreneurship,” and “Understand,” indicating a clear orientation toward contextual studies. Within this cluster, which is the largest in the network, several examples of these objectives can be highlighted, such as: Analyzing how social and solidary entrepreneurship is configured in Ecuador as a viable alternative to boost local development (Méndez et al., 2022); Analyzing the structural, social, and economic challenges of Mexican nano-enterprises in contexts of vulnerability (Lagunas et al., 2021); Understanding the entrepreneurial ecosystem in Latin American intercultural settings as a basis for regional and local development policies (Pitre Redondo, 2021); and Understanding how worldview and social imaginaries influence Mokaná cultural entrepreneurship and its relationship with public policies (Jiménez-Reyes, 2021).

**Figure 7:**
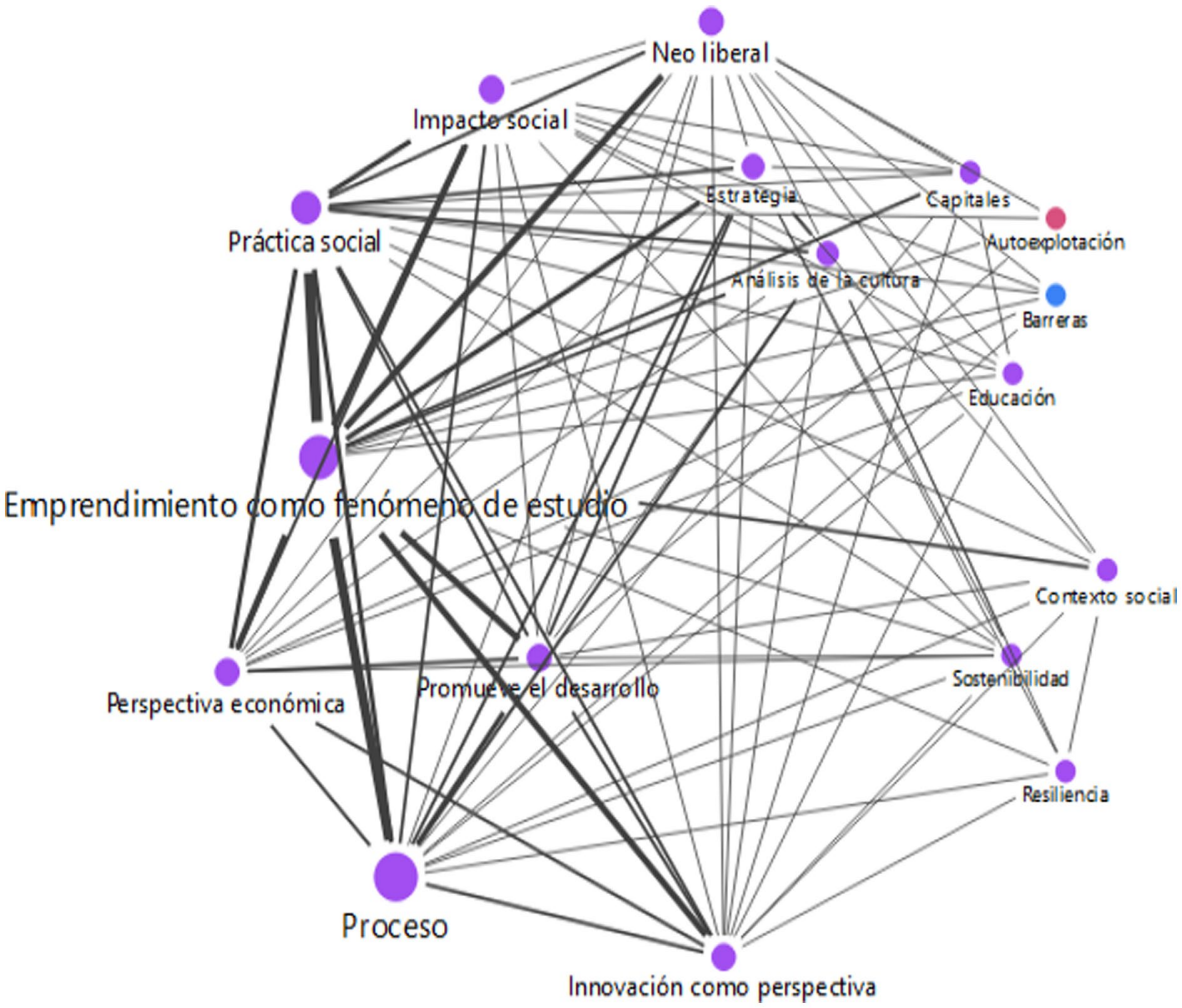
Semantic association network from the perspective of the concept of entrepreneurship as a phenomenon object of study in sociology. Source: own elaboration.

This perspective is reinforced by the frequent association of entrepreneurship with notions such as social context, relationships, and development models, suggesting that research tends to frame it as a collective practice situated within relational webs. This finding dialogues directly with the predominance of the group level in the analytical frameworks of the reviewed articles, where entrepreneurship is primarily addressed through network dynamics, organizational bonds, and community experiences. Thus, an approach is configured that recognizes social interactions as key mediators of entrepreneurship, although they are not always intentionally articulated with broader structural dimensions Figure 7.

On the other hand, the semantic network constructed from the conceptual perspectives of the analyzed articles allows us to observe how entrepreneurship is understood in recent Latin American literature. The cluster with the greatest centrality and density of connections where entrepreneurship is the focal phenomenon includes the notions of “process” and “social practice.” This configuration indicates that, far from being understood as a punctual or strict economic act, entrepreneurship is primarily conceived as a dynamic, situated, and relational process. As stated in several studies, entrepreneurship is conceived as a collective process oriented towards solving social problems and building social capital through non-profit organizational structures (Duarte-Alfonso, 2024). Likewise, it is understood as a dynamic process of economic and social value creation based on innovation, support networks, and public policies that foster its development (Palacios Trujillo, 2024).

The analysis suggests that the studies do not reduce the phenomenon to functionalist categories but rather approach it through frameworks that recognize its inscription within specific historical, cultural, and political contexts. Entrepreneurship is seen as an economic and social driver, key to subsistence and improving quality of life, yet affected by structural barriers and a lack of access to credit (Mejía, 2023). It is an activity that combines social and individual factors to generate products, services, and income, essential for innovation and social development (Menegaz, 2023). Thus, the idea of entrepreneurship is reinforced not as an end, but as a form of situated action that expresses adaptations, disputes, and meanings in societies marked by inequality.

**Figure 8:**
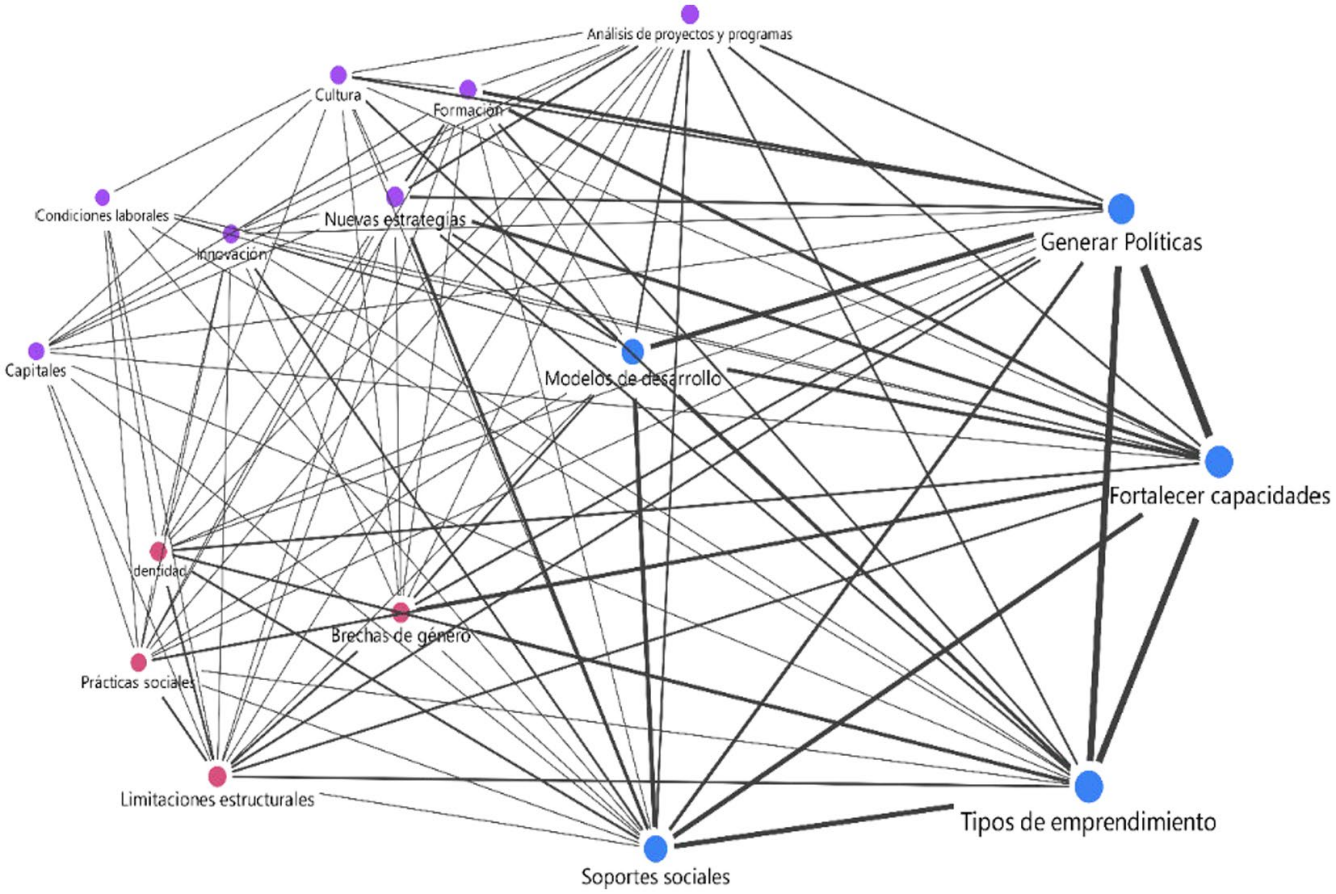
Semantic association network on the main findings and conclusions of the articles analyzed. Source: own elaboration.

Finally, within this framework, a critical reading of the phenomenon is evident, especially in the proximity between the “neoliberal” concept a constant critique and “social impacts,” which relates discourses of self-efficacy and personal achievement to “capitals” (referring to their absence or the need for strengthening) and self-exploitation as a management model for the activity. This idea is clearly expressed by Gambarotta (2025, p. 1), who states: “The entrepreneur’s path is perceived as a fun adventure that sets us free and is within anyone’s reach, inside a capitalist society for which there is no alternative.” Political and journalistic discourse consolidates an ideal that naturalizes individual competitiveness and depoliticizes social critique Figure 8.

The network reveals a high density of connections among clusters representing concepts such as “inequality,” “precariousness,” “autonomy,” “gender,” “popular economy,” and “resistance.” These associations demonstrate that the conclusions of the reviewed studies tend to problematize entrepreneurship beyond its economic dimension, highlighting it as a response to adverse structural conditions. The recurring presence of terms such as “informality,” “exclusion,” and “neoliberalism” reaffirms the critical lens that guides much of literature: entrepreneurship is not simply a path for individual development, but a situated strategy for social reproduction in contexts marked by the lack of formal employment, public policy cuts, and the commodification of common goods.

**Figure 9:**
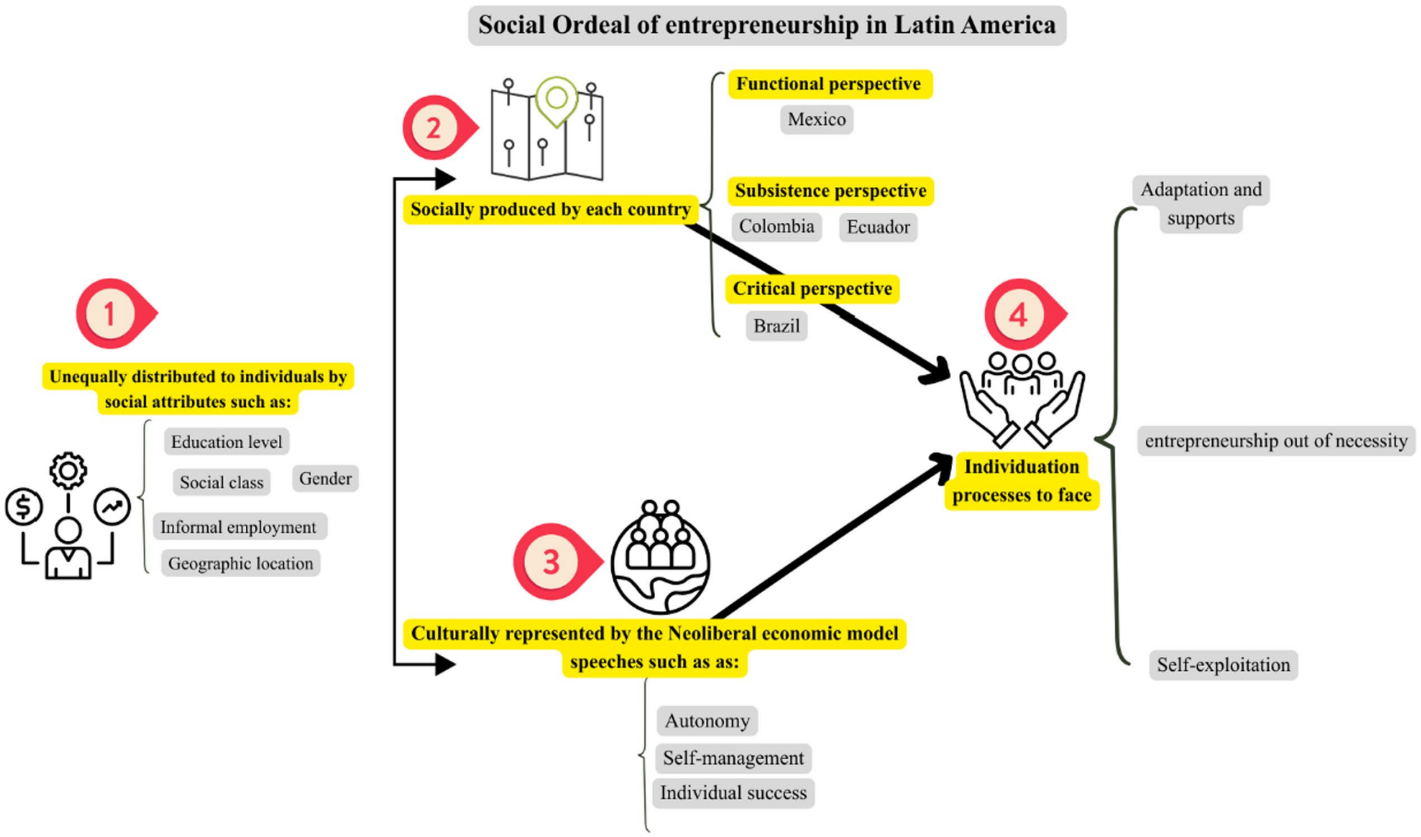
Analysis model: the social ordeal of entrepreneurship in Latin America. Source: own elaboration.

Likewise, concepts such as “resilience,” “collective,” “solidarity,” and “alternatives” appear interconnected. This infers that many investigations conclude by valuing entrepreneurial experiences as forms of organizational resistance or the construction of alternative economies. This aligns with the dominant group-level analysis previously identified and reinforces the idea that entrepreneurship in Latin America is largely configured as a collective, adaptive, and relational phenomenon, rather than an expression of competitive individualism Figure 9.

## Discussion

From left to right, the figure exemplifies a model that explains entrepreneurship in Latin America as a social ordeal based on the patterns identified across the analyzed articles. Social ordeals are inscribed in a temporal and diverse manner, evolving throughout an individual’s life course. As individuals age, they accumulate the outcomes achieved across various ordeals, particularly those concerning school, work, and family. Consequently, it is possible to speak of a set of standardized ordeals which, when analyzed, are capable of describing a specific socio-historical Landscape (Martuccelli, 2013a,b).

First, this ordeal is unequally distributed by attributes the individual does not choose social class, geographic location, or the baseline of labor informality, as well as others constructed throughout life, such as educational level. Second, it is evident how each country socially constructs this notion of an ordeal; at least from academic reading since sociology there are three perspectives on the phenomenon emerge: a critical perspective, a functional one, and another centered on subsistence, in the figure you can observe the main representative countries in each line according to the amount of articles and the analysis made in the results section. Third, it is culturally represented within the framework of the neoliberal economic model, that promotes specific discourse that guides the social behavior of the entrepreneur, giving them a big and alone responsibilities to consolidate the business forward. This is linked with cultural beliefs around the phenomenon that normally bring low social recognition the entrepreneur activity.

Fourth, the previous three factors configure the individuation process of the entrepreneur. This means how the individual faces in his daily life that structural challenges, the strategies for adaptation and permanent consolidation of support, mainly familiar and in his own self-management, explain the reasons for maintaining the activity of entrepreneurship despite the structural disadvantages. Consequently, Martuccelli (2013a,b) argues that a sociological approach must be capable of understanding the perceived distance between the actor’s expectations which generally remain unfulfilled and reality.

Ultimately, the sociology of modernity must achieve a new and specific reflection on the relationship between the individual and the world they inhabit, moving away from the notion of a total and harmonious world that has never existed. Research on entrepreneurship can focus on the singularity of the individual, which, according to Martuccelli (2013a), lies in understanding the very existence of each life as a particular mode of access to the world. This is what ensures that, beyond shared situations, an individual remains specific and distinctive from others, while emphasizing the need to maintain the link between the individual and the structural dimensions of society. As stated by Bautista and Joya (2023); Parra and Gutkind (2023), the sociology of individuation seeks to approach the great problems of society through the prism of individual singularity. It is a sociology at the scale of individuals that integrates the macro sociological ambition to explain society as a whole.

The analysis presented in the model of social ordeal identified patterns reveals that entrepreneurship in Latin America is not a response to market dynamism but rather a paradox of agency within precariousness. While motivation by opportunity predominates in developed economies, regional data show that the emergence of new productive units is, a symptom of developing economies facing significant challenges in establishing formal systems to absorb the labor force. Sociologically, this explains why high rates of entrepreneurial activity coincide with low levels of innovation and sustainability: entrepreneurship acts as a social escape valve, where individual agency is forced to manage survival in the absence of institutional support.

The interconnection between informality, inequality, and neoliberalism configures an ecosystem of self-exploitation within a management framework that depends entirely on the individual. Under neoliberal rationality, a discourse of autonomy has been naturalized, transferring all risk responsibility to the individual and legitimizing precariousness under the label of “entrepreneurial spirit.” This dynamic is exacerbated by structural inequality, which segments opportunities by social class, gender, and geography, pushing vulnerable sectors toward an informality that is not transitory but a way of life. Thus, informality is not an external failure but the territory where the individual negotiates their existence against a model that promotes competition while maintaining unreachable barriers to access.

In this sense, entrepreneurship is an intersectional phenomenon that transcends the economic sphere. Entrepreneurship is not limited to an economic necessity; it also responds to personal motivations linked to self-actualization and the socio-cultural context (Texis Flores et al., 2023). In the same line (Pérez, 2022) explains that female entrepreneurship at the border is constructed not only as an economic outlet but also as a space for autonomy and empowerment Women face structural gender limitations but leverage social and family networks to support business development in Latin America.

The review reveals an epistemological fracture in the region: while production in Brazil is profoundly critical, that of Mexico tends to be more functional. In Brazil, entrepreneurship is analyzed as a manifestation of contemporary “Americanism” and the “uberization” of work, denouncing the erosion of labor rights under the ideology of self-management. Conversely, Mexican academia focuses on the resilience and adaptation of small businesses, analyzing how social and human capital allows subjects to navigate post-pandemic uncertainty. This difference suggests that while Brazil theorizes entrepreneurship as a neoliberal mechanism of social control, Mexico approaches it as a necessary, albeit precarious, tool for subsistence and economic agency.

In Colombia and Ecuador, the dominant pattern links entrepreneurship to survival amidst the precariousness of formal employment, where 51% of workers in the region operate in informality. Here, inequality interconnects with the lack of access to fair financing, forcing individuals to assume individual risks to compensate for state shortcomings. Thus, entrepreneurship ceases to be an autonomous choice and becomes a form of “mandatory individuation,” where the subject must be an “entrepreneur of self” out of sheer structural necessity. As Romero-Bernal (2023) shows for Colombian case that the policy of the ‘orange economy’ constitutes a social control strategy that promotes docile subjectivities responsible for their own success or failure. The articles emphasize the socially conditioned and politically charged nature of entrepreneurship, making it a practice that must be understood through both its capacity for agency and its links to power structures, inequality, and exclusion.

Based on this, the conceptualization of entrepreneurship in Latin America must be deepened from an empirical and theoretical sociological perspective. Consequently, it is essential to promote studies that approach entrepreneurship as a social ordeal or proof in the sense proposed by contemporary sociological theory: a practice where individual agency is configured in permanent tension and dialogue with structural conditions. Studies should focus on analyzing the way in which individuals confront, as ordeals, the structural conditions of their existence, so the fundamental role of sociology consists in linking individual experiences with macro-social dynamics, demonstrating that what people experience on a personal level is intimately connected to and shaped by broader structural configurations (Martuccelli, 2010).

As last point, the traditional conception of the Entrepreneurial Ecosystem must be resinified under the light of the sociology of modernity. In Latin America, the ecosystem does not function as a harmonic network of institutional support, but rather as the complex and often hostile stage where the structural ordeal of entrepreneurship is enacted. While the literature distinguishes between opportunity and necessity-based entrepreneurship, this binary is insufficient to capture the depth of the phenomenon. From the perspective of mandatory individuation, the “push” factors informality, violence, and institutional fragility are not mere economic indicators; they are the specific conditions that force the individuation processes.

In this sense, the transition from necessity to opportunity is not just a change in business metrics, but a manifestation of the singularity of the individual. It represents a particular mode of access to the world where the entrepreneur, through tactical resilience, negotiates the distance between unfulfilled social expectations and a precarious reality. Therefore, analyzing entrepreneurship at the scale of individuals allows us to understand how the ecosystem rather than providing a safety net transfers systemic risks to the subject, transforming the act of creating a business into a definitive test of socio-historical survival.

## Conclusion

Entrepreneurship in Latin America must cease to be analyzed as a purely administrative phenomenon and instead be understood as a social ordeal of individuation. The findings confirm that, within the core of neoliberal rationality, the individual is compelled to undertake entrepreneurship not as a path of innovation, but as an adaptive response to the precariousness of formal employment. This unequally distributed ordeal detaches the State from the individual, forcing an autonomy that, in practice, typically results in the management of one’s own vulnerability and precariousness.

The primary methodological contribution of this study lies in the implementation of a Mixed Methods Systematic Review (MMSR), which enabled the triangulation of bibliometric trends with discursive patterns. Theoretically, this research contributes an integrative model that bridges the gap between individual agency and macro-structural constraints. By identifying the plural epistemological production, this study provides a novel framework for understanding entrepreneurship as a process of mandatory individuation, offering a critical lens to rethink how to support the entrepreneurship and entrepreneurs understanding the cultural and social specific contexts. This conclusion invites to develop empirical studies that promote a deep understanding of the social ordeal and individuation process that could explain the comprehension between structural factors and specific life trajectories.

The use of mixed methods is the necessary path to resolve the current limitations in the field. While quantitative studies often remain on the surface of business creation frequencies, and qualitative ones within the individual anecdote, the integration of both allows for capturing the process of individuation. Only through data triangulation can it be made visible how macro-tensions (neoliberalism/inequality) materialize in micro-decisions (life trajectories), enabling a holistic understanding that separate methods otherwise fragment.

Regarding future lines of study, the results suggest that public policies to promote entrepreneurship must be accompanied by a strengthening of support structures. If entrepreneurship remains a response to necessity rather than opportunity, the risk of business mortality and social exclusion will persist. It is necessary to create a model that protects the individual entrepreneur, mitigating the solitude of the structural ordeal through collaborative networks and social guarantees that collectivize the risk that the individual currently assumes in isolation.

## Data Availability

The original contributions presented in the study are included in the article/Supplementary material, further inquiries can be directed to the corresponding author.
